# Reducing the metabolic cost of walking with an ankle exoskeleton: interaction between actuation timing and power

**DOI:** 10.1186/s12984-017-0235-0

**Published:** 2017-04-27

**Authors:** Samuel Galle, Philippe Malcolm, Steven Hartley Collins, Dirk De Clercq

**Affiliations:** 1 Department of Movement and Sports Sciences, Ghent University, Watersportlaan 2, 9000 Gent, Belgium; 2 Department of Biomechanics and Center for Research in Human Movement Variability, University of Nebraska at Omaha, 6160 University Dr S, Omaha, NE 68182 USA; 3 Department of Mechanical Engineering, Carnegie Mellon University, 5000 Forbes Avenue, Pittsburgh, PA 15213 USA

**Keywords:** Human locomotion, Augmentation, Lower-limb exoskeletons, Metabolic cost, Optimal assistance

## Abstract

**Background:**

Powered ankle-foot exoskeletons can reduce the metabolic cost of human walking to below normal levels, but optimal assistance properties remain unclear. The purpose of this study was to test the effects of different assistance timing and power characteristics in an experiment with a tethered ankle-foot exoskeleton.

**Methods:**

Ten healthy female subjects walked on a treadmill with bilateral ankle-foot exoskeletons in 10 different assistance conditions. Artificial pneumatic muscles assisted plantarflexion during ankle push-off using one of four actuation onset timings (36, 42, 48 and 54% of the stride) and three power levels (average positive exoskeleton power over a stride, summed for both legs, of 0.2, 0.4 and 0.5 W∙kg^−1^). We compared metabolic rate, kinematics and electromyography (EMG) between conditions.

**Results:**

Optimal assistance was achieved with an onset of 42% stride and average power of 0.4 W∙kg^−1^, leading to 21% reduction in metabolic cost compared to walking with the exoskeleton deactivated and 12% reduction compared to normal walking without the exoskeleton. With suboptimal timing or power, the exoskeleton still reduced metabolic cost, but substantially less so. The relationship between timing, power and metabolic rate was well-characterized by a two-dimensional quadratic function. The assistive mechanisms leading to these improvements included reducing muscular activity in the ankle plantarflexors and assisting leg swing initiation.

**Conclusions:**

These results emphasize the importance of optimizing exoskeleton actuation properties when assisting or augmenting human locomotion. Our optimal assistance onset timing and average power levels could be used for other exoskeletons to improve assistance and resulting benefits.

**Electronic supplementary material:**

The online version of this article (doi:10.1186/s12984-017-0235-0) contains supplementary material, which is available to authorized users.

## Background

Walking is the most frequent means of human locomotion [1]. While humans use many strategies to reduce energy expenditure [2], walking still requires a considerable amount of metabolic energy, sometimes referred to as the ‘metabolic cost’ of walking. Assisting the ankle joint with an exoskeleton can reduce the metabolic cost of walking to below the cost of normal walking [3–6]. This shows that it is possible to reduce metabolic cost through robotic assistance.

Reductions in the metabolic cost of walking with ankle-foot exoskeletons result from two competing factors. A benefit can be derived from the exoskeleton when it acts to assist gait, expressed as the difference between powered exoskeleton^1^ walking and walking in zero-work mode^1^. However, wearing the exoskeleton in zero-work mode typically results in a metabolic penalty, expressed as the difference between normal walking^1^ without an exoskeleton and walking in zero-work mode. Some full-body exoskeletons have resulted in large metabolic penalties (e.g. [7]) while lightweight ankle-foot exoskeletons have resulted in penalties of less than 3% for active autonomous^1^ exoskeletons [4] and even close to zero for passive autonomous^1^ exoskeletons [5]. Reducing the penalty of wearing an exoskeleton in zero-work mode is mainly a design challenge, while increasing the difference between the zero-work condition and powered exoskeleton conditions is mainly a biomechanics challenge.

In order to solve the latter human-exoskeleton interaction challenge, optimal assistance properties (e.g. actuation timing, assistance magnitude, etc.) are crucial to further reduce the metabolic energy cost of walking. Malcolm et al. [3] showed that the timing of exoskeleton actuation onset (referred to as actuation timing) is an important exoskeleton property that influences the metabolic cost of walking with active exoskeletons. They found a convex landscape in metabolic cost versus actuation timing with an optimum around 40% of the stride. Studies that have found the highest reductions in metabolic energy cost have also used an actuation timing around 40% of the stride [4, 6].

Of course, actuation timing is not the only determinant of metabolic cost when walking with ankle-exoskeleton assistance. Assistance magnitude also seems to have a strong effect [8, 9]. The average positive mechanical exoskeleton power per stride summed for both ankles (referred to here as exoskeleton power) can be as high as 0.38 W∙kg^−1^ resulting in reductions in net metabolic cost of between 10 and 22% for powered exoskeleton conditions compared to zero-work conditions [3, 4, 6, 8, 10–12]. However, comparing these studies does not result in a clear relationship between exoskeleton power magnitude and metabolic cost, likely because many factors differ between studies (e.g. design, exoskeleton mass, actuation profile, etc.), confounding comparisons. The simplest walking model [13] would suggest that increasing exoskeleton power will reduce the mechanical energy requirements for walking until subjects walk with zero metabolic cost. Indeed, a recent study, in which both ankle and hip joints were assisted with a soft exo-suit [8] indicated that metabolic energy cost reduces linearly with increasing exoskeleton assistance magnitude, similar to some findings with active prostheses [9]. On the other hand, a study on unilateral exoskeleton assistance suggested an exponential relationship between device power and metabolic cost [14]. Experiments and simulation studies with exoskeletons have similarly suggested that under some conditions “more is not always better” [5, 15, 16]. Interpretations have been made more difficult by the limited range of attainable levels of exoskeleton power, which has often been between 50 and 80% of biological ankle power [4, 10, 11].

In order to study if and when the reduction in the metabolic cost of walking begins to level-off with increasing exoskeleton power during bilateral exoskeleton assistance, it seems necessary to deliver more power than in current studies. To identify the influence of exoskeleton power magnitude on the metabolic cost of walking, as well as the interaction with actuation timing, there is a need for a parametric study of actuation timing and exoskeleton power over a larger range. A study of both actuation characteristics is also expected to contribute to an improved understanding of the assistive mechanisms of ankle-foot exoskeletons. Several studies have indicated that other joints besides the ankle joint are involved in the reduction in metabolic cost experienced when using an ankle-foot exoskeleton [3–5, 8, 12, 17, 18] but the exact mechanisms are unclear. Exploring different assistance parameters over a broad range would help to identify the relationship between biomechanical changes and the resulting changes in metabolic cost.

The overall goal of this study was to characterize the relationship between ankle exoskeleton power, actuation timing, and metabolic cost during walking over a broad range. We used a tethered and powered plantarflexion-assisting exoskeleton to vary actuation onset timing and average exoskeleton power independently and over a broad range and studied the influence of these characteristics on the metabolic energy cost of walking. We expected a second-order effect of actuation timing on metabolic energy cost [3] and explored several candidate relationships between exoskeleton power and metabolic energy cost to evaluate the interaction between timing, power and metabolic cost. A secondary goal was to use the best relationship to define optimal assistance parameters. Finally, we analyzed muscle activation, exoskeleton kinetics and walking kinematics that describe the neuromechanical interaction between the exoskeleton and the human, with the goal of explaining the reduction in metabolic cost and improving our understanding of human-exoskeleton interaction.

## Methods

### Subjects

Fourteen female subjects participated in the experiment from which ten were retained (age 23 ± 1.2 y; weight 61.0 ± 4.5 kg; height 168.1 ± 5.2 cm; European shoe size 38.6 ± 0.8). Non-inclusions were due to technical failure of the exoskeleton (two subjects), drop-out (one subject) and errors in data synchronization (one subject). We selected female subjects of normal height and weight to be able to use one exoskeleton size for all subjects and to achieve relatively large amounts of exoskeleton power normalized to bodyweight. None of the subjects had prior experience using an exoskeleton. All participants provided written informed consent prior to participation. The experimental protocol was approved by the ethical committee of the Ghent University Hospital.

### Exoskeleton

**Fig. 1:**
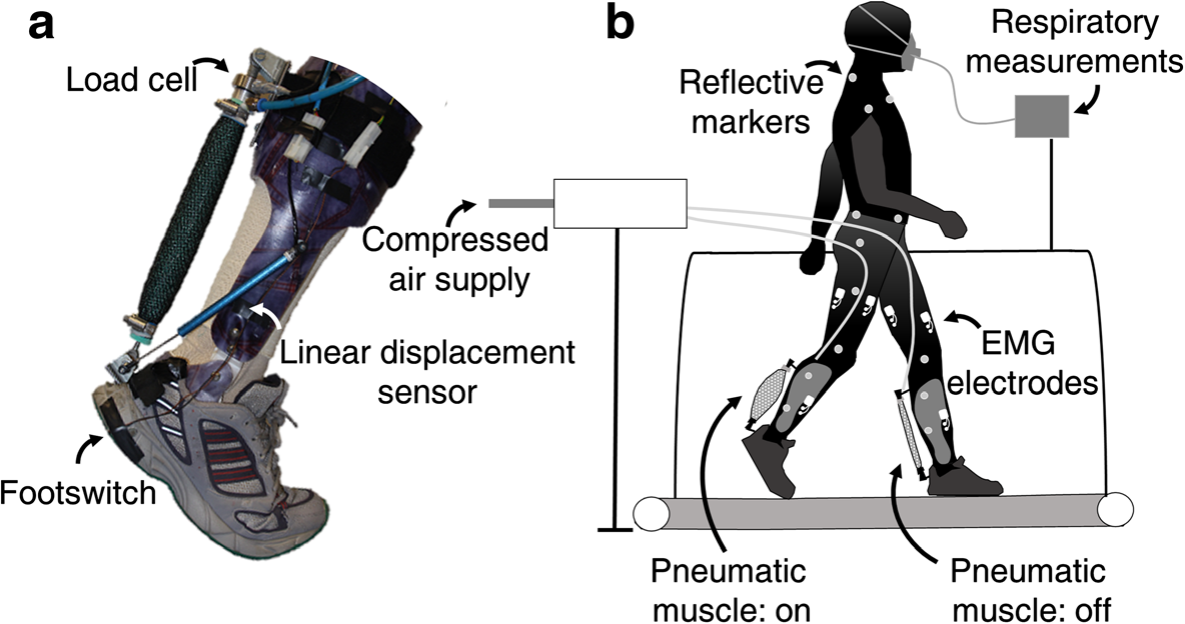
Schematic representation of the experimental set-up. The exoskeleton (**a**) has footswitches in the heel to detect foot contact and a load cell that is mounted proximal to the pneumatic muscle to measure tension. A linear displacement sensor that is connected between the foot segment and the shank segment measures ankle joint angle for real-time control. During the experiment subjects wore an exoskeleton at each leg (**b**) and walked on a treadmill. The pneumatic muscles were activated during ankle push-off and assisted plantarflexion. Subjects wore EMG electrodes, reflective markers and a face mask for data collection. Not all reflective markers and EMG electrodes are represented in the figure

The bilateral exoskeleton (Fig. 1) consisted of an ankle-foot orthosis at each leg with a hinge at the ankle joint and pneumatic muscles [3, 17–19]. The pneumatic muscles were 0.27 m in length and were connected between the foot segment and the shank segment. They ‘contracted’ when inflated with compressed air. The locations of the pneumatic muscles insertions were individually adjusted such that they allowed 15° of dorsiflexion when passive. The exoskeleton fitted inside standard sport shoes. Footswitches (Multimec 5E/5G, Mec, Ballerup, Denmark) were built in to detect foot contact. Load cells (100 Hz; 210 Series, Richmond Industries Ltd., Reading, United Kingdom) were connected between the orthoses and the pneumatic muscles to measure the force of the pneumatic muscles. Linear displacement sensors (100 Hz; SLS130, Penny&Giles, Christchurch, United Kingdom) were connected between the foot and shank sections of the exoskeletons to measure ankle joint angles [20]. The total weight of one tethered exoskeleton including all sensors was 0.890 kg (0.680 kg for the orthosis, 0.030 kg for the displacement sensor, 0.110 kg for the pneumatic muscle and 0.070 kg for the load cell). Additional hardware, including an air supply, was placed next to the treadmill.

### Actuation timing and exoskeleton power

Based on footswitch signals from the previous stride, exoskeleton actuation for the next stride was controlled using fixed percentages of stride time with a feedforward algorithm in Labview (National Instruments, Austin, TX, USA) [3, 17–19]. To control the amount of exoskeleton mechanical power, air pressure in the pneumatic muscles was adjusted using an iterative learning algorithm [9, 21]. For exoskeleton real-time control, average positive exoskeleton ankle joint mechanical power was measured in real-time using the load cell and the linear displacement sensor (Fig. 1). Based on a prior calibration with motion capture, ankle joint angle and moment arm of the pneumatic muscles were estimated based on linear displacement [20]. A moving average of positive exoskeleton power with a window of 10 strides was then used as the input for the iterative learning algorithm. Over the course of many steps, the algorithm slowly increased or decreased air pressure to the pneumatic muscles when average power was too low or too high, respectively, in order to achieve a desired amount of average exoskeleton power during walking.

### Experimental conditions

**Fig. 2:**
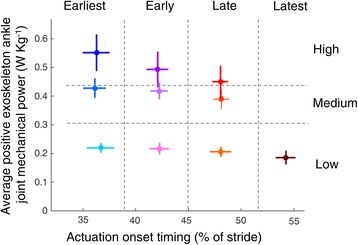
Parameter sweep of actuation timing and exoskeleton power. Actuation timing and exoskeleton power for the 10 powered exoskeleton conditions tested. *Dots* are population averages and *lines* are standard deviations. Each condition resulted in a distinct combination of timing and power

The experiment consisted of a habituation session and a data collection session with roughly one week in between. A total of 14 conditions were applied in each session: a NormalWalking condition, in which subjects walked with normal shoes without an exoskeleton; a ZeroWork condition, in which subjects walked with the exoskeleton but without assistance from the pneumatic muscles; and 12 powered exoskeleton conditions. The 12 powered conditions were based on a two dimensional parameter sweep of four actuation onset timings (actuation timings) and three average positive exoskeleton ankle joint mechanical power levels (exoskeleton powers) (Fig. 2). Four timing values where applied with actuation onset timing of 36 ± 1%, 42 ± 1%, 48 ± 1% and 54 ± 1% of the stride (referred to as Earliest, Early, Late and Latest, respectively) and with actuation always ending at 64 ± 1% of the stride. These values were chosen to be close to the optimal actuation onset timing from a previous study [3]. Three exoskeleton power levels were applied, with values of 0.21 ± 0.02 W∙kg^−1^, 0.41 ± 0.03 W∙kg^−1^ and the maximum achievable amount with our exoskeleton and control methods, which was 0.50 ± 0.06 W∙kg^−1^ (referred to as Low, Medium and High, respectively). These values were chosen based on the capabilities of our exoskeleton testbed, and coincided with approximately 50, 100 and 125% of the net biological ankle work observed during normal walking [4, 10, 11]. This exceeded the largest range of prior exoskeleton studies [8]. It was not possible to apply more than 0.2 W∙kg^−1^ with Latest timing due to bandwidth limitations of the pneumatic actuators. Lower amounts of power with this timing did not appear to be useful. We therefore chose to include only 10 powered exoskeleton conditions in our analysis: three actuation timings (Earliest, Early and Late) for which three power levels were applied (Low, Medium and High) and a fourth actuation timing (Latest) for which only one power level was applied (Low) (Fig. 2).

### Experimental protocol

In the habituation session, subjects learned to walk with the exoskeleton and became accustomed to the experimental set-up. They walked in all powered conditions, the ZeroWork condition and the NormalWalking condition on the treadmill at 1.25 m∙s^−1^. All conditions lasted three minutes with two minutes of rest in between. Conditions were applied in random order, apart from the NormalWalking condition which was always first or last due to the time to don and doff the exoskeleton.

Experimental data were collected on a separate day, about a week later. Each subject performed the same walking protocol as in the habituation session, but conditions lasted for four minutes to reach steady-state metabolic rate. A standing rest condition in which subjects stood still for four minutes was also applied before the walking conditions to capture resting metabolic rate. Air pressure of the pneumatic muscles at the beginning of each powered condition was set to be the same as the air pressure from the end of each powered condition in the habituation session, such that the iterative learning algorithm did not need to change air pressure substantially during powered exoskeleton conditions on the data collection day.

### Data collection

Subjects wore a face mask connected to a gas analysis system that measured O_2_ consumption and CO_2_ production continuously (Cosmed, K4b2, Rome, Italy) (Fig. 1). Exoskeleton sensors (footswitches, displacement sensors and load cells) also measured continuously during the entire experiment (Fig. 1). Full body 3D kinematics were recorded with 51 reflective markers (four on each foot, two on each exoskeleton foot segment, two on each exoskeleton ankle joint, six on each exoskeleton shank segment, two on each knee joint, four on a plate connected to each thigh, six on the pelvis, and five on the torso) and 14 infrared cameras (200 Hz; Pro Reflex, Qualisys AB, Gothenburg, Sweden) with Qualisys software. Surface electromyography (EMG) of the m. (Musculus) soleus, m. gastrocnemius medialis, m. tibialis anterior, m. vastus lateralis, m. rectus femoris, m. biceps femoris and m. gluteus maximums of both legs were measured with bipolar surface electrodes and wireless transmitters (1000 Hz; ZeroWire, Noraxon, Scottsdale, AZ, USA). Electrodes were placed in accordance with SENIAM guidelines [22]. For the m. soleus and m. gastrocnemius medialis, holes were cut in the orthosis on the approximate locations. Marker data and surface EMG data were collected for 10s during the last minute of each condition, which included around nine full strides.

### Data processing

Metabolic energy cost of walking was the primary study outcome. It was estimated based on O_2_ consumption and CO_2_ production during the last two minutes of each condition using a standard equation [23]. Net metabolic cost was calculated by subtracting the metabolic cost of standing at rest from gross metabolic cost during walking and was normalized to body weight.

All time series data presented in figures were time normalized from heel contact to the next heel contact of the same leg, averaged for the left and right leg and averaged across subjects to calculate population stride averages.

Kinematics were analyzed to evaluate the effect of exoskeleton assistance on walking patterns. An eight segment model (two feet, two shanks, two thighs, one pelvis and one torso) was used to calculate sagittal plane joint angles with Visual 3D software (C-Motion, MD, USA). Dempster’s regression equations [24] were used to define segment masses, with some alterations: the mass of the arms and head were added to the torso and the mass of the foot and shank sections of the exoskeleton were added to the foot and shank segments, respectively, of the model for the exoskeleton conditions. Heel contact and toe-off were automatically detected using foot kinematics [25]. Toe-off timing was expressed as a percentage of the stride time. Time between consecutive heel contacts was multiplied with treadmill speed to calculate step length.

Exoskeleton kinetics were calculated to assess actuation timing and exoskeleton power. Marker positions and pneumatic muscle force data were filtered with a Butterworth low-pass filter with a cut-off frequency of 12 Hz. Ankle joint angle and moment arm of the pneumatic muscles were calculated using motion capture. Ankle joint angular velocity was calculated as the first derivative of the ankle joint angle in time. Moment arm of the pneumatic muscle force was calculated frame-by-frame as the minimum distance between the ankle joint axis and the pneumatic muscle centroid. Pneumatic muscle force, measured via a load cell, was multiplied with the moment arm to calculate exoskeleton torque and multiplied with ankle joint angular velocity to calculate instantaneous exoskeleton mechanical power. Actuation onset timing was calculated based on the maximum of the second derivative of the unfiltered exoskeleton torque, which provides a robust measure of torque onset [21]. Exoskeleton power was calculated as the numerical integration of positive instantaneous exoskeleton power over the stride, divided by the stride time and summed for both legs. We chose to sum average exoskeleton power for both legs in order to facilitate comparisons to the reduction in body metabolic rate resulting from assistance.

EMG data were measured to describe the neuromuscular interaction between the exoskeleton and the user. Surface EMG data were band pass filtered (50–450 Hz) and rectified. A moving root mean square with a window of 100 ms was then applied. All EMG values were normalized to the peak value of the ZeroWork condition so that the peak value in the ZeroWork condition represents a value of 100. For all muscle groups, peak values were calculated for instances where a clear activation burst was present in the stride. For the m. tibialis anterior this was the peak value in the beginning of the stance phase (between 1 and 62% of the stride) and in the beginning of the swing phase (between 62 and 80% of the stride). For the m. soleus this was the peak value in the stance phase (between 1 and 62% of the stride) and in the beginning of the swing phase (between 62 and 80% of the stride). For the m. gastrocnemius medialis this was the peak value during the stance phase (between 1 and 62% of the stride). For the m. rectus femoris this was the peak value in the stance phase (between 1 and 62% of the stride) and the peak value around toe-off (between 40 and 80% of the stride). For the m. biceps femoris this was the peak value during the swing phase (between 62 and 100% of the stride). For the m. vastus lateralis and the m. gluteus maximus this was the peak value during the stance phase (between 1 and 62% of the stride).

Center-of-mass power, which is calculated by the dot product of center-of-mass velocity and ground reaction force, can be used to distinguish four functional phases of stance during walking: collision, rebound, pre-load and push-off [26, 27]. In prior studies, the rebound phase has been observed to covary with metabolic cost [5, 8, 9, 28–30]. Rebound work is calculated as the area under the positive burst of center-of-mass power during the single stance phase. It represents the positive center-of-mass work that is done by leg straightening, possible elastic rebound of the knee, and hip work that moves the swing leg [26]. We calculated center-of-mass velocity and acceleration by taking the first and second derivative of center-of-mass position in the treadmill belt reference frame. Ground reaction force was estimated based on center-of-mass acceleration and body mass. This rough approximation of the ground reaction force can replace the direct ground reaction force measurement when no force plate is available [31]. While data from double support could not be usefully interpreted [32], it was possible to retain center-of-mass power during the rebound phase (positive burst during the single stance phase) and to calculate average rebound power as the numerical integration of rebound power, divided by stride time. We expressed average rebound power in W∙kg^−1^.

### Data analysis

To evaluate steady state in metabolic rate measurements, a repeated measures analysis of variance (ANOVA) was performed between sequential 30s averages from the four minute walking conditions with post-hoc paired *t*-tests (SPSS Statistics 21, IMB, Armonk, NY, USA). Other statistical analyses were performed with Matlab (MathWorks, Natick, MA, USA). Repeated measures ANOVA (*P* ≤ 0.05) were performed to establish differences in metabolic cost, step-time parameters, EMG metrics and center-of-mass metrics between all conditions. When a statistical difference was found between conditions, pairwise comparisons were performed using post-hoc paired *t*-tests (*P* ≤ 0.05) to search for differences between the powered exoskeleton conditions (*n* = 10) and the ZeroWork condition and between the powered exoskeleton conditions (*n* = 10) and the NormalWalking condition. We used a Šídák-Holm correction for multiple testing [33] for these 20 comparisons. We did not compare the 10 powered exoskeleton conditions with each other using paired *t*-tests. Instead, when significant differences were found between one or more powered exoskeleton conditions and the ZeroWork condition or between one or more powered exoskeleton conditions and the NormalWalking condition, we used a regression analysis to express changes in specific outcome parameters resulting from varying actuation timing and power magnitude levels.

A two-dimensional regression was used to describe overall trends in outcome parameters as the result of changes in actuation timing and power magnitude. The reduction in metabolic energy cost compared to the zero-work condition was chosen as the dependent variable and actuation timing and exoskeleton power were chosen as independent variables. This regression analysis was done based on the population averages but we also calculated individual regressions to illustrate individual differences (Additional file 1: Figure S1). The regression analysis was done for 14 cases (10 powered exoskeleton conditions and four ZeroWork conditions), where the Zerowork condition was assessed at each timing. Hence, in the ZeroWork condition the exoskeleton power equals zero, which was added for the average of every actuation timing (Earliest, Early, Late, Latest) so that the surface fit would include the ZeroWork condition for all actuation timings. Coefficients of determination (R^2^) were calculated to evaluate how well the curve fits matched with the 10 powered conditions and the four ZeroWork conditions. We performed regressions to several candidate formulae (Table 1). In each formula we included a second-order relationship between the reduction in metabolic cost and actuation timing based on previous research [3]. This second-order relationship was then multiplied by exoskeleton power, reflecting the expectation that timing would not affect outcomes when power was zero. For the independent relationship between exoskeleton power and metabolic cost we considered three additional terms: (1) a linear relationship, (2) a second-order relationship and (3) an exponential relationship. These candidate terms were based on conflicting observations from previous studies [3, 8, 9, 14]. This resulted in three candidate formulae:

**Table 1:** Regression analysis results

	Formula	R^2^	*P*
(1)	Δ*E* = − 0.096 + 9.2 ⋅ *Pavg* − 0.49 ⋅ *Ton* ⋅ *Pavg* + 0.0055 ⋅ *Ton* ^2^ ⋅ *Pavg*	0.833	0.01
(2)	Δ*E* = 0.0088 + 9.1 ⋅ *Pavg* + 5 ⋅ *Pavg* ^2^ − 0.64 ⋅ *Ton* ⋅ *Pavg* + 0.0077 ⋅ *Ton* ^2^ ⋅ *Pavg*	0.990	<0.001
(3)	Δ*E* = − 10 + 10 ⋅ *exp* ^(0.89 ⋅ *Pavg*)^ *Pavg* − 0.62 ⋅ *Ton* ⋅ *Pavg* + 0.0075 ⋅ *Ton* ^2^ ⋅ *Pavg*	0.988	<0.001

Results of three different regressions that were used as potential candidates to result in a good fit for the effect of actuation timing and exoskeleton power on metabolic cost of exoskeleton walking with the resulting R^2^ and *P*-values

1 $$ \Delta E = a+ b \cdot Pavg+ c\cdot T o n\cdot Pavg+ d\cdot T o{n}^2\cdot Pavg $$

2 $$ \Delta E = a+ b \cdot Pavg+ c\cdot P a v{g}^2+ d\cdot T o n\cdot P a v g+ e\cdot T o{n}^2\cdot P a v g $$

3 $$ \Delta E = a+ b \cdot ex{p}^{\left( c\cdot Pavg\right)} Pavg+ d\cdot T o n\cdot Pavg+ e\cdot T o{n}^2\cdot Pavg $$

Where *ΔE* is the change in net metabolic cost compared to the ZeroWork condition (e.g. 0.51 W∙kg^−1^); *Ton* is actuation onset (e.g. 43%); and *Pavg* is exoskeleton power (e.g. 0.2 W∙kg^−1^).

We identified the coefficients for each formula that resulted in the best fit, i.e. the least residual sum of squares (Table 1). The coefficients for regression 2 and 3 resulted in very similar coefficients of determination (R^2^). Regression 2, with a second order relationship for both the actuation timing and exoskeleton power determinants, resulted in a slightly higher R^2^ value. While this difference is negligible and not sufficient to prefer one of both regressions, all High exoskeleton power conditions resulted in a higher metabolic cost compared to the Medium power conditions (Fig. 4. and Additional file 4: Figure S4). This suggests that there is not only a levelling-off in the metabolic cost when high power magnitude levels are present but that there is even an optimal exoskeleton power magnitude in our results. As such, we decided to use regression 2, with a second order relationship for both the actuation timing and exoskeleton power determinants, for further analysis. Following the metabolic cost, the same fitting method was then used to relate EMG data to actuation timing and exoskeleton power:

$$ \mathrm{E}\mathrm{M}\mathrm{G}\ \mathrm{metric} = a+ b \cdot Pavg+ c\cdot P a v{g}^2+ d\cdot T o n\cdot P a v g+ e\cdot T o{n}^2\cdot P a v g $$

The same analysis was done to relate center-of-mass data to actuation timing and exoskeleton power:

$$ \mathrm{Center}\hbox{-} \mathrm{of}\hbox{-} \mathrm{mass}\kern0.5em \mathrm{metric} = a+ b \cdot Pavg+ c\cdot P a v{g}^2+ d\cdot T o n\cdot P a v g+ e\cdot T o{n}^2\cdot P a v g $$

Statistical tests for the effect of actuation timing and exoskeleton power on metabolic cost, EMG metrics and center-of-mass average rebound power were performed through this regression analysis (*P* ≤ 0.05).

## Results

**Fig. 3:**
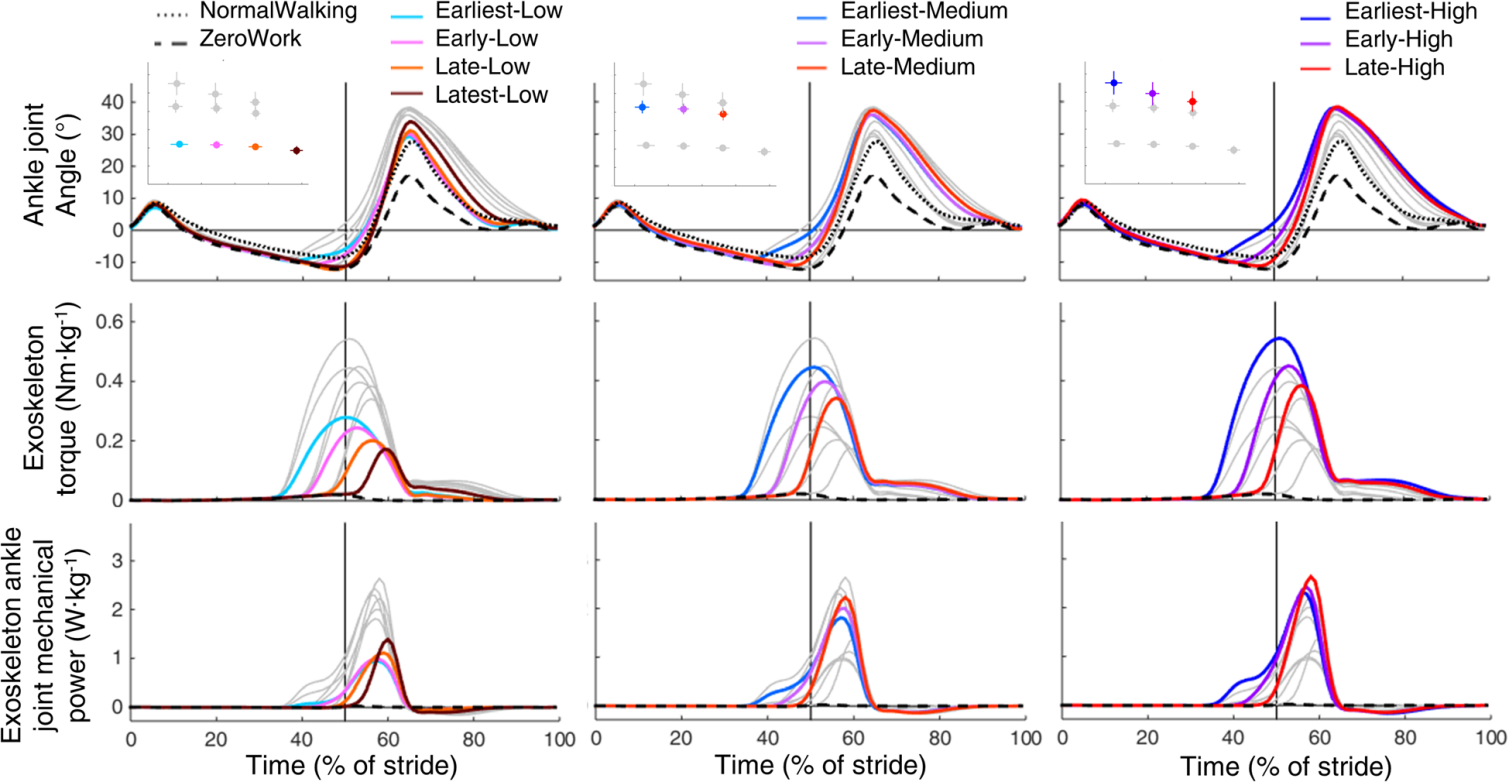
Ankle joint angle, exoskeleton torque and exoskeleton power. Population averages, normalized from heel contact to heel contact for ankle joint angle, exoskeleton torque and exoskeleton ankle joint mechanical power of the 10 powered conditions, the ZeroWork condition and the NormalWalking condition. All powered conditions are shown in *grey*, with selected conditions in *color*. Subplots at top show which conditions are plotted in color in each panel. The *vertical grey line* represents opposite heel contact

Subjects walked in 10 powered exoskeleton conditions (based on combinations of the four different actuation timings (Earliest, Early, Late, Latest) and three different exoskeleton power levels (Low, Medium, High)), a ZeroWork condition and a NormalWalking condition. The distribution of actuation timing and exoskeleton power shows that the desired parameter space was successfully covered (Fig. 2) and that the powered conditions were clearly distinguishable from each other. Earlier actuation timings resulted in an earlier onset of pneumatic muscle force (Additional file 2: Figure S2) and an earlier onset of exoskeleton torque (Fig. 3). Increased air pressure in the pneumatic muscles (Additional file 2: Figure S2) increased exoskeleton peak torque in conditions with more exoskeleton power (Fig. 3). This resulted in an earlier plantarflexion onset with earlier actuation timings and higher peak plantarflexion angles in conditions with more exoskeleton power (Fig. 3). Step length (*P* = 0.01) and toe-off timing (*P* = 0.01) showed small differences between conditions but were very similar overall. Step length was 0.68 ± 0.01 m on average across all conditions and toe-off timing occurred at 62 ± 1% of stride on average across all conditions.

**Fig. 4:**
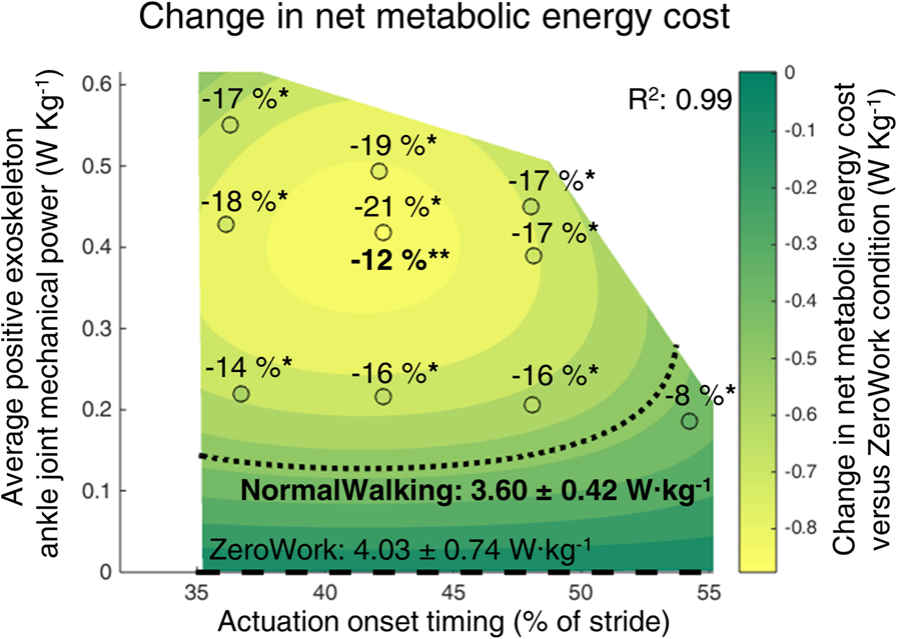
Net metabolic energy cost. Net metabolic cost is depicted versus actuation timing and average positive exoskeleton power. Metabolic cost for the powered exoskeleton conditions is expressed as the reduction versus the ZeroWork condition. Repeated measures ANOVA indicated significant differences in net metabolic energy cost between conditions (*P* < 0.001). * indicates a statistically significant reduction versus ZeroWork and ** indicates a statistically significant reduction versus NormalWalking based on post-hoc *t*-tests with Šídák-Holm correction (*P* ≤ 0.05). The *percentages above* each condition are the statistically significant reductions in net metabolic cost versus the ZeroWork condition. The *percentages underneath* each condition (in bold) are the statistically significant reductions in net metabolic cost versus NormalWalking. The *surface gradient* is the result of a two-dimensional regression for which R^2^ is provided (*P* < 0.001). The *black dotted line* indicates the metabolic cost for NormalWalking and the *black dashed line* shows the ZeroWork condition with the absolute net metabolic energy cost given in numbers for both conditions

Subjects’ metabolic cost reached steady state in the last two minutes of each walking condition (Additional file 3: Figure S3). A significant difference was found for net metabolic cost between conditions (*P* < 0.001). Net metabolic cost of all powered conditions was significantly lower than that in the ZeroWork condition (4.03 ± 0.74 W∙kg^−1^) (Fig. 4 and Additional file 4: Figure S4). The lowest net metabolic cost was found in the Early-Medium condition (3.16 ± 0.55 W∙kg^−1^), with a reduction of 21.4 ± 5.6% compared to ZeroWork and 12.3 ± 9.3% compared to NormalWalking (3.60 ± 0.74 W∙kg^−1^). In the Early-Medium condition, actuation onset was 42.3 ± 0.8% of stride and average power was 0.42 ± 0.03 W∙kg^−1^. The metabolic penalty of wearing the exoskeleton in ZeroWork compared to NormalWalking was 11.6 ± 9.6%. Although the metabolic cost of powered exoskeleton walking was lower in almost all conditions compared to NormalWalking, only the difference in the Early-Medium condition was statistically significant (Fig. 4 and Additional file 4: Figure S4). The ratio of the change in metabolic rate to exoskeleton mechanical power for the Early-Medium condition, was 2.1 ± 0.8. Higher ratios were found in the Early-Low and Late-Low conditions, with values of 3.1 ± 0.9 and 3.1 ± 1.4, respectively. Earlier or later timings or higher amounts of exoskeleton power resulted in lower ratios.

Regression analyses established strong relationships between actuation timing (Ton), exoskeleton power (Pavg) and the reduction in metabolic cost (ΔE). Consistent with the choice for a quadratic relationship for both actuation timing and exoskeleton power (Table 1), metabolic cost was higher in all Low and High conditions compared to the corresponding Medium conditions and metabolic cost was higher in all Earliest and Late conditions compared to the Early condition (Fig. 4 and Additional file 4: Figure S4). The resulting regression for the metabolic cost was statistically significant (*P* <0.001) and predicted experimental values well (R^2^ = 0.99) (Table 1). This 2D non-linear model combining actuation timing and average exoskeleton power therefore provided a strong estimate of the reduction in metabolic cost of walking with this exoskeleton. The regression suggests metabolic cost is minimized when actuation timing is 41% and exoskeleton power is 0.41 W∙kg^−1^, close to the Early-Medium condition (Fig. 4). There did not appear to be substantial interactions between optimal timing and optimal power; an onset of 41% stride minimized cost for all power levels, and a power level of 0.41 W∙kg^−1^ minimized cost for all timings (Additional file 4: Figure S4). Individual regressions sometimes deviated from the average profile (Additional file 1: Figure S1).

**Fig. 5:**
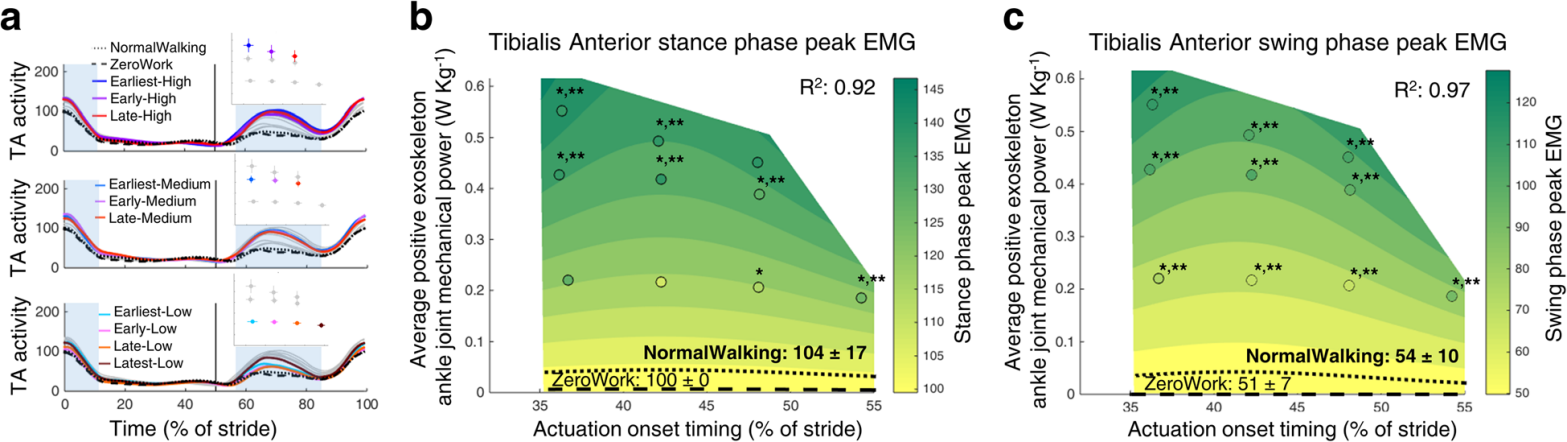
Electromyography of the m. tibialis anterior. Time series show population averages for EMG of the m. tibialis anterior (TA) averaged for the left and right leg and plotted from heel contact to heel contact (**a**). The *grey vertical line* represents opposite heel contact. All powered conditions are shown in *grey*, with selected conditions in *color*. Subplots at top show which conditions are plotted in color. *Blue rectangles* show the periods where a peak EMG value was analyzed. Repeated measures ANOVA indicated significant differences between conditions for the peak EMG in the beginning of the stance phase (between 1 and 62% of the stride; *P* < 0.001) and for the peak EMG in the beginning of the swing phase (between 62 and 80% of the stride; *P* < 0.001). The effect of actuation timing and exoskeleton power on peak EMG in the beginning of the stance phase (**b**) and the beginning of the swing phase (**c**) is indicated by a regression analysis. The *surface gradient* is the result of a two-dimensional regression for which R^2^ is provided for the peak EMG during the stance phase (*P* = 0.002) and during the swing phase (*P* < 0.001). The *black dotted line* shows the NormalWalking condition and the *black dashed line* shows the ZeroWork condition with the absolute value for both conditions. * indicates a statistically significant reduction versus ZeroWork and ** indicates a statistically significant reduction versus NormalWalking based on post-hoc *t*-tests with Šídák-Holm correction (*P* ≤ 0.05)

**Fig. 6:**
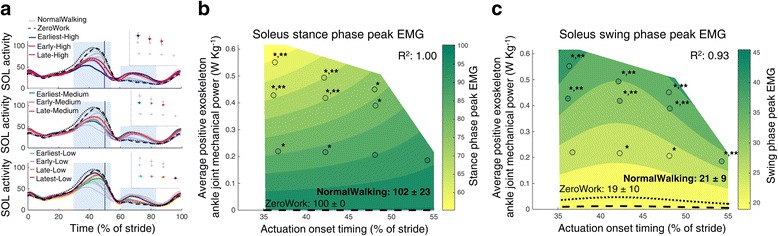
Electromyography of the m. soleus. Time series show population averages for EMG of the m. soleus (SOL) averaged for the left and right leg and plotted from heel contact to heel contact (**a**). The *grey vertical line* represents opposite heel contact. All powered conditions are shown in *grey*, with selected conditions in *color*. Subplots at top show which conditions are shown in color. *Blue rectangles* show the periods where a peak EMG value was analyzed. Repeated measures ANOVA indicated significant differences between conditions for the peak EMG in the stance phase (between 1 and 62% of the stride; *P* < 0.001) and the peak EMG in the beginning of the swing phase (between 62 and 80% of the stride; *P* < 0.001). The effect of actuation timing and exoskeleton power on peak EMG in the beginning of the stance phase (**b**) and the beginning of the swing phase (**c**) is indicated by a regression analysis. The *surface gradient* is the result of a two-dimensional regression for which R^2^ is provided for the peak EMG during the stance phase (*P* < 0.001) and during the beginning of the swing phase (*P* < 0.001). The *black dotted line* shows the NormalWalking condition and the *black dashed line* shows the ZeroWork condition with the absolute value for both conditions. *indicates a statistically significant reduction versus ZeroWork and ** indicates a statistically significant reduction versus NormalWalking based on post-hoc *t*-tests with Šídák-Holm correction (*P* ≤ 0.05)

**Fig. 7:**
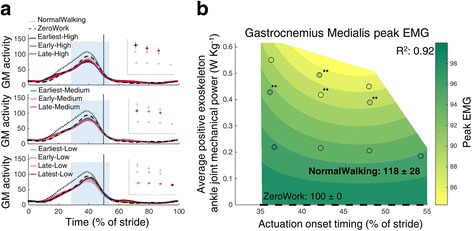
Electromyography of the m. gastrocnemius medialis. Time series show population averages for EMG of the m. gastrocnemius medialis (GM) averaged for the left and right leg and plotted from heel contact to heel contact (**a**). The *grey vertical line* represents opposite heel contact. All powered conditions are shown in *grey*, with selected conditions in *color*. Subplots at top show which conditions are shown in color. *Blue rectangles* show the periods where a peak EMG value was analyzed. Repeated measures ANOVA indicated significant differences between conditions for the peak EMG in the beginning of the stance phase (between 1 and 62% of the stride; *P* < 0.001). The effect of actuation timing and exoskeleton power on peak EMG in the beginning of the stance phase (**b**) is indicated by a regression analysis. The *surface gradient* is the result of a two-dimensional regression for which R^2^ is provided for the peak EMG during the stance phase (*P* < 0.001). The NormalWalking condition is not visible as the value for NormalWalking is situated out of the range for which the regression is shown but the value of the NormalWalking condition is shown. The *black dashed line* shows the ZeroWork condition with the absolute value. * indicates a statistically significant reduction versus ZeroWork and ** indicates a statistically significant reduction versus NormalWalking based on post-hoc *t*-tests with Šídák-Holm correction (*P* ≤ 0.05)

**Fig. 8:**
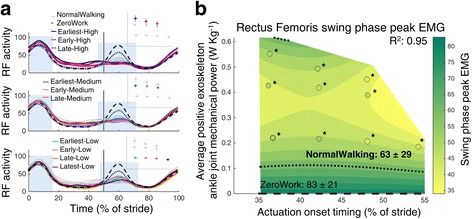
Electromyography of the m. rectus femoris. Time series show population averages for EMG of the m. rectus femoris (RF) averaged for the left and right leg and plotted from heel contact to heel contact (**a**). The *grey vertical line* represents opposite heel contact. All powered conditions are shown in *grey*, with selected conditions in *color*. Subplots at top show which conditions are shown in color. *Blue rectangles* show the periods where a peak EMG value was analyzed. No significant differences were found between conditions for the first EMG peak during the stance phase (between 1 and 62% of the stride; *P* = 0.23). Therefore, no regression analysis was done for this EMG metric. Repeated measures ANOVA indicated significant differences between conditions for the peak EMG around toe-off (between 40 and 80% of the stride; *P* < 0.001). The effect of actuation timing and exoskeleton power on peak EMG near toe-off (**b**) is indicated by a regression analysis. The *surface gradient* is the result of a two-dimensional regression for which R^2^ is provided (*P* < 0.001). The *black dotted line* shows the NormalWalking condition and the *black dashed line* shows the ZeroWork condition with the absolute value for both conditions. * indicates a statistically significant reduction versus ZeroWork and ** indicates a statistically significant reduction versus NormalWalking based on post-hoc *t*-tests with Šídák-Holm correction (*P* ≤ 0.05)

**Fig. 9:**
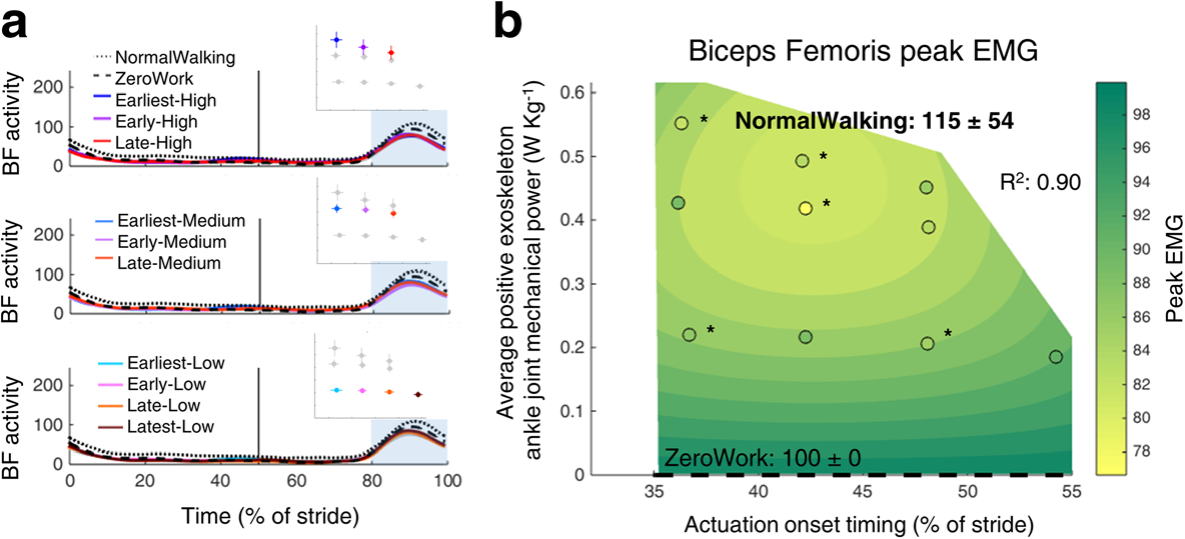
Electromyography of the m. biceps femoris. Time series show population averages for EMG of the m. biceps femoris (BF) averaged for the left and right leg and plotted from heel contact to heel contact (**a**). The *grey vertical line* represents opposite heel contact. All powered conditions are shown in *grey*, with selected conditions in *color*. Subplots at top show which conditions are shown in color. *Blue rectangles* show the periods where a peak EMG value was analyzed. Repeated measures ANOVA indicated significant differences between conditions for the peak EMG in the swing phase (between 62 and 100% of the stride; *P* < 0.001). The effect of actuation timing and exoskeleton power on peak EMG in the swing phase (**b**) is indicated by a regression analysis. The *surface gradient* is the result of a two-dimensional regression for which R^2^ is provided for the peak EMG during the swing phase (*P* < 0.001). The NormalWalking condition is not visible as the value for NormalWalking is situated out of the range for which the regression is shown but the value of the NormalWalking condition is shown. The *black dashed line* shows the ZeroWork condition with the absolute value. * indicates a statistically significant reduction versus ZeroWork and ** indicates a statistically significant reduction versus NormalWalking based on post-hoc *t*-tests with Šídák-Holm correction (*P* ≤ 0.05)

**Fig. 10:**
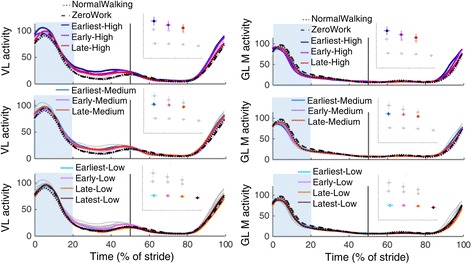
Electromyography of the m. vastus lateralis and m. gluteus maximus. Time series show population averages for EMG of the m. vastus lateralis (VL) and the m. gluteus maximus (GL M) averaged for the left and right leg and plotted from heel contact to heel contact. The *grey vertical line* represents opposite heel contact. All powered conditions are shown in *grey*, with selected conditions in *color*. Subplots at top show which conditions are shown in color. *Blue rectangles* show the periods where a peak EMG value was analyzed. Repeated measures ANOVA indicated significant differences between conditions for the EMG peak during the stance phase (between 1 and 62% of the stride; *P* = 0.05) for the m. vastus lateralis, but post-hoc t-tests with Šídák-Holm correction did not show any significant difference between the powered conditions and the NormalWalking condition or the ZeroWork conditon. Therefore, no regression analysis was done for this EMG metric. Repeated measures ANOVA indicated no significant differences between conditions for the EMG peak during the stance phase (between 1 and 62% of the stride; *P* = 0.95) for the m. gluteus maximus. Therefore, no regression analysis was done for this EMG metric

Changes in patterns of muscle activity may help illustrate the function of specific muscles during walking and how their activity changes with exoskeleton assistance (Figs. 5, 6, 7, 8, 9 and 10). The tibialis anterior muscle showed a large increase in muscle activity at the beginning of the stance phase and at the beginning of the swing phase in the powered conditions (Fig. 5a). Peak EMG during the beginning of the stance phase was significantly higher for almost all Medium and High power conditions compared to ZeroWork and NormalWalking. The regression analysis indicated that higher amounts of exoskeleton power resulted in bigger increases in m. tibialis anterior EMG (Fig. 5b). Peak EMG in the beginning of the swing phase was significantly higher for all powered conditions compared to ZeroWork and NormalWalking, with increases of more than 100% in several powered conditions. Regression analysis suggested that more exoskeleton power resulted in higher m. tibialis anterior activity (Fig. 5c).

Exoskeleton assistance reduced m. soleus activity during ankle push-off but seemed to increase EMG activity during the swing phase (Fig. 6). Peak muscle activity in the stance phase was reduced by more than 30% compared to ZeroWork and NormalWalking with early actuation onset and high exoskeleton power. The regression analysis indicated that m. soleus activity was most reduced in conditions with early actuation timings and high amounts of power. Soleus activity in the beginning of the swing phase increased slightly, possibly the result of crosstalk with the tibialis anterior muscle [34], as not much m. soleus activity is expected in this phase of the gait cycle. This is further suggested by the similarity between the regression analysis for both m. soleus and m. tibialis anterior peak EMG activity during the swing phase. During push-off however, tibialis anterior muscle activity was negligible and the measured signal probably originated only from the soleus muscle.

Peak gastrocnemius medialis EMG during push-off seemed to be reduced compared with both the NormalWalking and the ZeroWork condition (Fig. 7). However, differences with the ZeroWork condition were not statistically significant. Several powered conditions showed peak EMG activity that was more than 20% lower than in the NormalWalking condition. Regression analysis showed that peak m. gastrocnemius EMG was most reduced with late actuation timings and high amounts of power.

Activity in the m. rectus femoris showed a peak in the beginning of the stance phase for all conditions and in the beginning of the swing phase in the NormalWalking and ZeroWork conditions. No significant differences were found for peak EMG between conditions in the beginning of the stance phase (*P* = 0.23). Peak EMG activity was substantially reduced in the swing phase (Fig. 8) by as much as 40% compared to ZeroWork for all conditions. However, differences between powered conditions were rather small, which was emphasized by the shape of the regression which did not show a clear effect of actuation timing and exoskeleton power.

Activity in the m. biceps femoris showed a peak in the end of the swing phase (Fig. 9). The EMG peak activity during the swing phase seemed lower in the powered conditions compared to the NormalWalking and ZeroWork conditions. Differences in peak EMG compared to the ZeroWork condition were only statistically significant in a few conditions. Regression analysis showed a strong relationship, with the highest reductions for intermediate timing values and Medium power levels.

Peak EMG activity during the stance phase in the m. vastus lateralis and the m. gluteus maximus did not show significant differences between the powered conditions and the NormalWalking or ZeroWork conditions (Fig. 10).

**Fig. 11:**
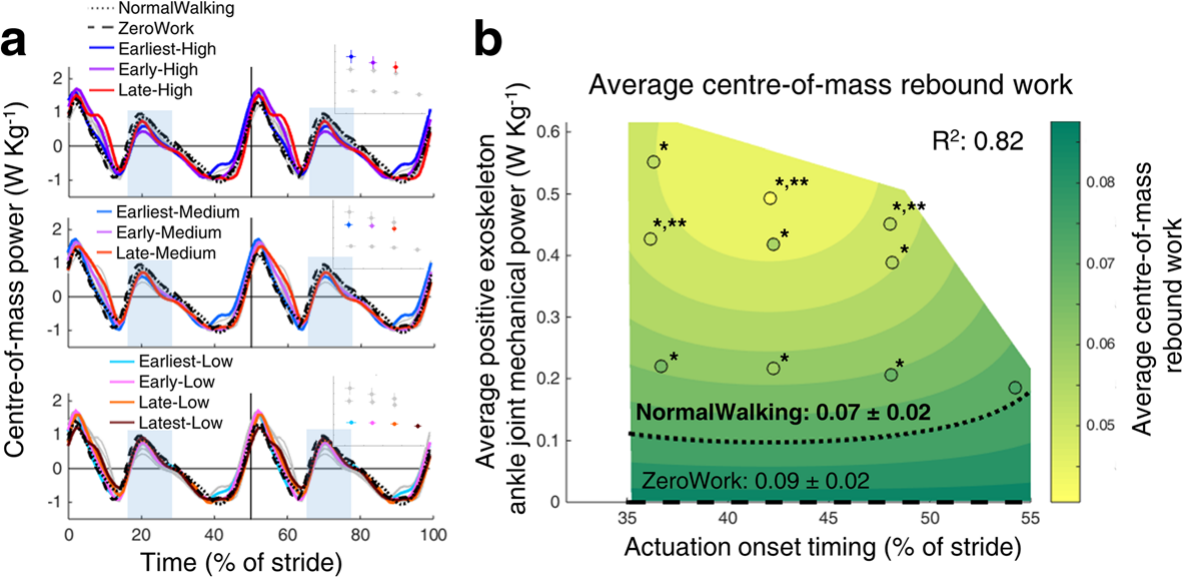
Center-of-mass power calculations. Time series show population averages for center-of-mass power plotted from heel contact to heel contact (**a**). The *grey vertical line* represents opposite heel contact. All powered conditions are shown in *grey*, with selected conditions in *color*. Subplots at top show which conditions are shown in color. *Blue rectangles* show the periods where the rebound phase was present and where the average center-of-mass power was calculated for the positive burst. Repeated measures ANOVA indicated significant differences between conditions for the average center-of-mass power during this rebound phase (*P* < 0.001). This was calculated as the numerical integration of rebound power, divided by stride time. The effect of actuation timing and exoskeleton power on average center-of-mass power is indicated by a regression analysis (**b**). The *surface gradient* is the result of a two-dimensional regression for which R^2^ is provided (*P* < 0.001). The *black dotted line* shows the NormalWalking condition and the *black dashed line* shows the ZeroWork value. * indicates a statistically significant reduction versus ZeroWork and ** indicates a statistically significant reduction versus NormalWalking based on post-hoc *t*-tests with Šídák-Holm correction (*P* ≤ 0.05)

Center-of-mass rebound power was significantly lower in almost all powered conditions compared to the ZeroWork condition. The regression analysis showed that average center-of-mass rebound work was reduced for high levels of exoskeleton power and early timings (Fig. 11) and had a pattern similar to that of the metabolic cost (Fig. 4).

## Discussion

The overall goal of this study was to determine the relationship between metabolic cost, exoskeleton actuation timing and exoskeleton power in an ankle-foot exoskeleton powered with pneumatic artificial muscles. We found strong effects of both terms, well-characterized by a two-dimensional quadratic function that suggested optimal actuation timing to be around 42% of stride and optimal average exoskeleton power to be around 0.4 W∙kg^−1^ summed for both legs (Fig. 4). One of our powered exoskeleton conditions, with actuation onset at 42.3 ± 0.8% of the stride and average exoskeleton power of 0.42 ± 0.03 W∙kg^−1^ summed for both legs, indeed resulted in a reduction in net metabolic cost of 21.4 ± 5.6% compared to walking with the exoskeleton without assistance from the pneumatic muscles. These reductions are similar to the highest reported results to date [8]. Benefits were offset by a penalty of 11.6 ± 9.6% for wearing the exoskeleton while inactive, a higher penalty than with some other recent exoskeletons [4, 5]. Optimal timing and power thereby reduced net metabolic cost by 12.3 ± 9.3% compared to normal walking, which is the highest reported reduction for an ankle exoskeleton compared to normal walking.

### Actuation timing

Our regression analysis indicated an optimal actuation onset of around 42% of the stride: when actuation timing was independently varied, metabolic cost followed a convex pattern. In each of the Low, Medium and High power conditions, metabolic cost had a U-shaped relationship with actuation timing (Fig. 4 and Additional file 4: Figure S4). Given that there did not seem to be an interaction effect with exoskeleton power, optimal actuation onset for an ankle exoskeleton with pneumatic muscles is around 42% of the stride. The optimal actuation timing that we found resulted from a trade-off between good m. soleus assistance (with higher reductions for an earlier timing), m. gastrocnemius EMG assistance (with higher reductions for a later timing) and low m. tibialis anterior EMG resistance (with smaller increases for an intermediate timing).

The actuation timing of the condition with the lowest metabolic cost, e.g. with actuation timing onset at 42.3 ± 0.8%, is close to the actuation timing with the lowest metabolic cost of our previous study [3]. Mooney et al. [4, 6] used a similar actuation timing for exoskeleton assistance, leading to high reductions in metabolic cost. The observed optimum is on the other hand later than the onset of torque in a passive exoskeleton with a mechanical clutch that reduced the metabolic cost of walking [5]. However, actuation with the exoskeleton in the present study delivers mostly positive power, whereas in the passive exoskeleton study the reduction in metabolic cost is (at least partly) attributed to eccentric torque support during ankle dorsiflexion in the single stance phase. Recently, Quinlivan et al. [8] also found large reductions in the metabolic cost with an earlier actuation onset timing. Their exoskeleton assisted both the ankle and the hip joint, which makes it difficult to compare results. Actuation torque ramped in more slowly with that device, such that the relatively low torques at the beginning of exoskeleton assistance may have had little effect on the user. Our observed optimum is on the other hand earlier than with a unilateral prosthesis emulator, where an optimum was found at 52% of stride or later [21]. This difference could be due to the differences between exoskeleton walking and prosthesis walking, the difference between unilateral and bilateral assistance, differences in the actuation profile or a combination of these factors.

### Average positive exoskeleton ankle power

Our regression analysis indicated an optimal amount of average exoskeleton power around 0.4 W∙kg^−1^ summed for both legs: when exoskeleton power was independently varied, metabolic cost followed a U-shaped pattern. The exoskeleton power of the condition with the lowest metabolic cost, e.g. with exoskeleton power of 0.42 ± 0.03 W∙kg^−1^ summed for both legs corresponds to around 100% of the net biological ankle joint work during walking without exoskeleton assistance [10, 11]. Higher levels of average exoskeleton power resulted in smaller reductions in metabolic cost, especially in the earlier actuation onset timing conditions. With an exo-suit that assisted both the ankle and the hip joints, an inverse linear relationship was found between exoskeleton power and the metabolic cost of walking until average net exoskeleton power was 0.19 W∙kg^−1^ per leg [8]. This seems in accordance with our results, with the addition that we found that higher amounts of exoskeleton power did not result in further reductions in the metabolic cost of walking. A similar trend was observed in a unilateral exoskeleton study, in which metabolic cost seemed to level off or worsen in the highest exoskeleton work conditions [9]. Other recent simulations and experiments [5, 15, 16] have found that ‘more is not always better’ for several parameters of walking (assistance), due to a trade-off between several factors that have a positive or a negative effect on the metabolic cost of walking. A similar trade-off seems present in our EMG results. For the m. soleus and the m. gastrocnemius we found that higher exoskeleton power was better but for the m. tibialis anterior high power was detrimental.

It must be mentioned that we chose a second order relationship over an exponential relationship in our regression analysis (Table 1). This choice was based on the fact that average metabolic cost was higher in all High conditions compared to the corresponding Medium conditions, which indicated that metabolic cost had a U-shaped relationship with exoskeleton power in each of the Earliest, Early and Late timing conditions (Fig. 4 and Additional file 4: Figure S4). Due to the small differences in exoskeleton power between our Medium and High conditions, it is difficult to draw strong conclusions. In prosthesis research [14, 35] an exponential relationship was found for the reduction in metabolic cost and average positive exoskeleton ankle power until at least 200% of the positive work that is delivered by the biological ankle joint during normal walking. It is hard to estimate how the relationship would extend when higher amounts of power are applied, especially in late actuation timings.

While we found an optimal amount of exoskeleton power around 0.4 W∙kg^−1^, lower amounts of power also resulted in a reduction in metabolic cost of 14 to 16% compared to walking in the zero-work mode. In these conditions, the ratio of change in metabolic rate to mechanical power was much higher than in the metabolically optimal condition. This suggests that the assistance in conditions with lower amounts of power (around 0.2 W∙kg^−1^) can be more efficient, which could be important for autonomous devices where battery weight is a concern.

### Assistive mechanism

The extensive parameter sweep that we performed improves overall insights into the assistive mechanism of ankle exoskeletons that result in a reduction in metabolic cost. Previous work suggested that exoskeletons could reduce step-to-step transition costs [3], which stem from the negative work performed in the collision phase and the positive work to compensate for this energy loss, both serving to redirect the center-of-mass from one arc to another [27]. Indeed, it has been shown that ankle exoskeletons replace some push-off work [4]. However, while some studies have found a reduction in contralateral collision during unilateral exoskeleton walking [9], other studies with unilateral exoskeletons and prostheses have not found a reduction in collision work [14, 21]. On a muscular level, we did not find reductions in activity of the m. gluteus maximus, the m. biceps femoris, the m. vastus lateralis or the m. rectus femoris during the collision phase, suggesting that costs related to collision were not reduced. Several studies have reported changes in metabolic cost in combination with changes in rebound work instead of collision work [5, 8, 9, 14, 28–30]. In this study we found a strong similarity between the optimal parameters for the reduction in metabolic cost and for reduction in rebound work. The rebound phase is often attributed to positive work of the stance leg and to contralateral leg swing [26, 36]. We found indications of assistance in leg swing initiation during exoskeleton assistance with a reduction in m. rectus femoris activity. One of the roles of the m. rectus femoris during the end of stance and the initiation of swing is to serve as a hip flexor to bring the swing leg forward [37]. This suggests less involvement of hip and knee musculature in leg swing during powered exoskeleton assistance. Several other studies have reported the influence of ankle assistance on the knee and hip joints [4, 12, 17, 18]. Similar findings were found in a prosthesis study [14], where ankle push-off work also implicated in assisting leg swing initiation. While we only assisted the ankle joint, it is likely that the increased plantarflexion and the higher plantarflexion velocity during push-off with exoskeleton assistance could influence the following leg swing. This seems to coincide with findings of Lipfert et al. [38], that linked the impulsive ankle push-off in walking to initiation of leg swing. As such, it seems that increased plantarflexion is an essential proviso for assisting leg swing initiation with an ankle exoskeleton. Other studies with exoskeletons that reduced metabolic cost and reported reduced knee and hip work also showed increases in plantarflexion [4, 12, 18]. This could also explain why other exoskeletons that imposed similar torque profiles but maintained normal joint angles did not result in a reduction in the metabolic cost of walking [39]. It may therefore not be optimal to emulate the biological ankle kinematics.

### Future work

Recent findings from simulations and experiments [5, 40] suggest that the metabolic cost of the plantarflexors is not only related to the push-off but also to isometric activity earlier in the stance phase. For example, reductions in metabolic cost of up to 7% are possible from torque support during the beginning of the stance phase [5]. A combination of replacing ankle joint work during push-off, assisting leg swing initiation (as a direct result of ankle assistance or by assisting the hip joint itself [41, 42]) like we did in our study and torque support during the beginning of stance, as was done in the passive exoskeleton study [5], could potentially lead to reductions in the metabolic cost of more than 25%, especially because midstance torque support can lead to inefficient, rapid shortening of plantarflexor muscles during push-off [5]. Exoskeletons that can vary torque and power in a more controlled way [43] could be used to manipulate the exoskeleton power profile and further focus on the combined assistance of torque support during stance and positive power assistance during push-off.

Because of the complexity of human locomotion, reducing the metabolic cost of walking with an exoskeleton is not straightforward. Due to the effect of exoskeleton assistance on walking pattern (e.g. [3]), on balance [44], on muscle activity (e.g. [10]), on elastic stretch and recoil (e.g. [5]), on fascicle power (e.g. [45]) and many other factors, it is difficult to predict human behavior resulting from exoskeleton assistance. One way to explore this space is to perform parameter sweep experiments in combination with supporting measures to understand why a specific condition is beneficial (e.g. [3, 8, 9, 14, 21]). These parameter sweep studies will remain necessary in the future to establish optimal assistance as the optimum will likely result from a combination of factors. Another promising approach is direct optimization of metabolic rate using human-in-the-loop optimization techniques [46–48]. At the moment experimental approaches are still preferred, as intuition is often deceiving and simulations are not yet very reliable [49] because of the complex human-exoskeleton interface. However, experimental results from studies like this one can be used to improve musculoskeletal models.

### Limitations

Our exoskeleton testbed was tethered to off-board hardware and power sources, while other exoskeletons have shown reductions in metabolic cost for autonomous devices where all the hardware and power sources were carried by the user [5, 6]. The large difference that we found between powered exoskeleton walking and exoskeleton walking in zero-work mode suggests that if we could apply our actuation profile with a lightweight autonomous exoskeleton [4–6, 8], reductions of more than 15% versus normal walking are possible. One possibility might be to rely on the human joints to serve as hinges, which shows promise [4, 6, 8].

Despite the recent introduction of autonomous exoskeletons [5, 6, 8] our exoskeleton testbed is still useful as it allows manipulation of exoskeleton assistance parameters in a broad range, which is important to improve the understanding of human-exoskeleton interaction. There is still a need for studies combining metabolic measurements, EMG, kinematics and kinetics during exoskeleton walking in a broad range of conditions to elaborate on these findings. Similar studies have improved insight into exoskeleton work and torque support [8, 9], unilateral prosthesis walking [14] and bilateral prosthesis walking [21].

One of the limitations of this study is that individual subjects exhibited differences in their responses to exoskeleton assistance. We searched for one optimal actuation timing and optimal exoskeleton power, while this sometimes seemed to differ between subjects. By using individual optimizations, it may be possible to further reduce metabolic cost. We found reductions of 26 ± 4% for powered versus ZeroWork walking and of 17 ± 10% for powered walking versus normal walking if we averaged the highest reductions from each subject.

We were unable to fully control the shape of the exoskeleton power curve and it is possible that other actuation parameters affect metabolic cost more strongly. For example, our exoskeleton testbed was not able to deliver sufficient power to elaborate the latest onset timing thoroughly. It would be useful to explore higher amounts of exoskeleton power with late timing. A future approach could be to evaluate the effect of the shape of the actuation profile on the metabolic cost of exoskeleton walking.

## Conclusions

This study examined the independent effects of ankle exoskeleton actuation timing and average exoskeleton power on the metabolic cost of walking. We showed that reductions in metabolic cost of 21% versus walking with the exoskeleton in zero-work mode and 12% versus normal walking without the exoskeleton are possible with optimal actuation timing and power. Actuation timing showed an optimum at 42% of the stride and average exoskeleton power was optimal at around 0.42 W∙kg^−1^. The assistive mechanisms leading to these reductions include reducing muscular activity of the plantarflexors during push-off and more proximal muscles during leg swing initiation.

## Additional files

Additional file 1: Figure S1. Change in net metabolic energy cost for individual subjects. (TIF 3 mb)

Additional file 2: Figure S2. Pneumatic muscle air pressure and pneumatic muscle force. (TIF 2 mb)

Additional file 3: Figure S3. Individual net metabolic energy cost. (TIF 3 mb)

Additional file 4: Figure S4. Net metabolic cost. (TIF 1 mb)

## Abbreviations

EMG: Electromyography
m.: Musculus
NormalWalking: Walking without exoskeleton
Pavg: Average positive exoskeleton ankle joint mechanical power per stride summed for both legs
Ton: Actuation timing onset
ZeroWork: Walking with the exoskeleton without assistance from the pneumatic muscles
ΔE: Reduction in net metabolic cost versus the ZeroWork condition

## Footnotes

1
*Normal walking* is used to refer to walking without an exoskeleton. *Active* exoskeletons refer to exoskeletons which use external energy to assist walking while *passive* exoskeletons store and re-use energy which is generated by the user and thus do not need external energy input. *Powered walking* refers to walking with exoskeleton assistance (both for passive and active exoskeleton assistance). Walking with the exoskeleton without assistance of the device (e.g. with the assistive spring disengaged for passive devices or without active assistance for active devices) is referred to as walking with *zero-work* instead of ‘unpowered’ exoskeleton walking (e.g. [3, 10]) to avoid confusion when concerning passive exoskeletons [5]. *Tethered* exoskeletons refer to a connection with off-board hardware or power source. *Autonomous* exoskeletons refer to devices were all hardware and power sources are carried by the user.
