# Supplementary figures and images for: Reducing the metabolic cost of walking with an ankle exoskeleton: interaction between actuation timing and power
Source: J Neuroeng Rehabil. 2017 Apr 27;14:35. doi: 10.1186/s12984-017-0235-0 (PMC5408443; doi:10.1186/s12984-017-0235-0)

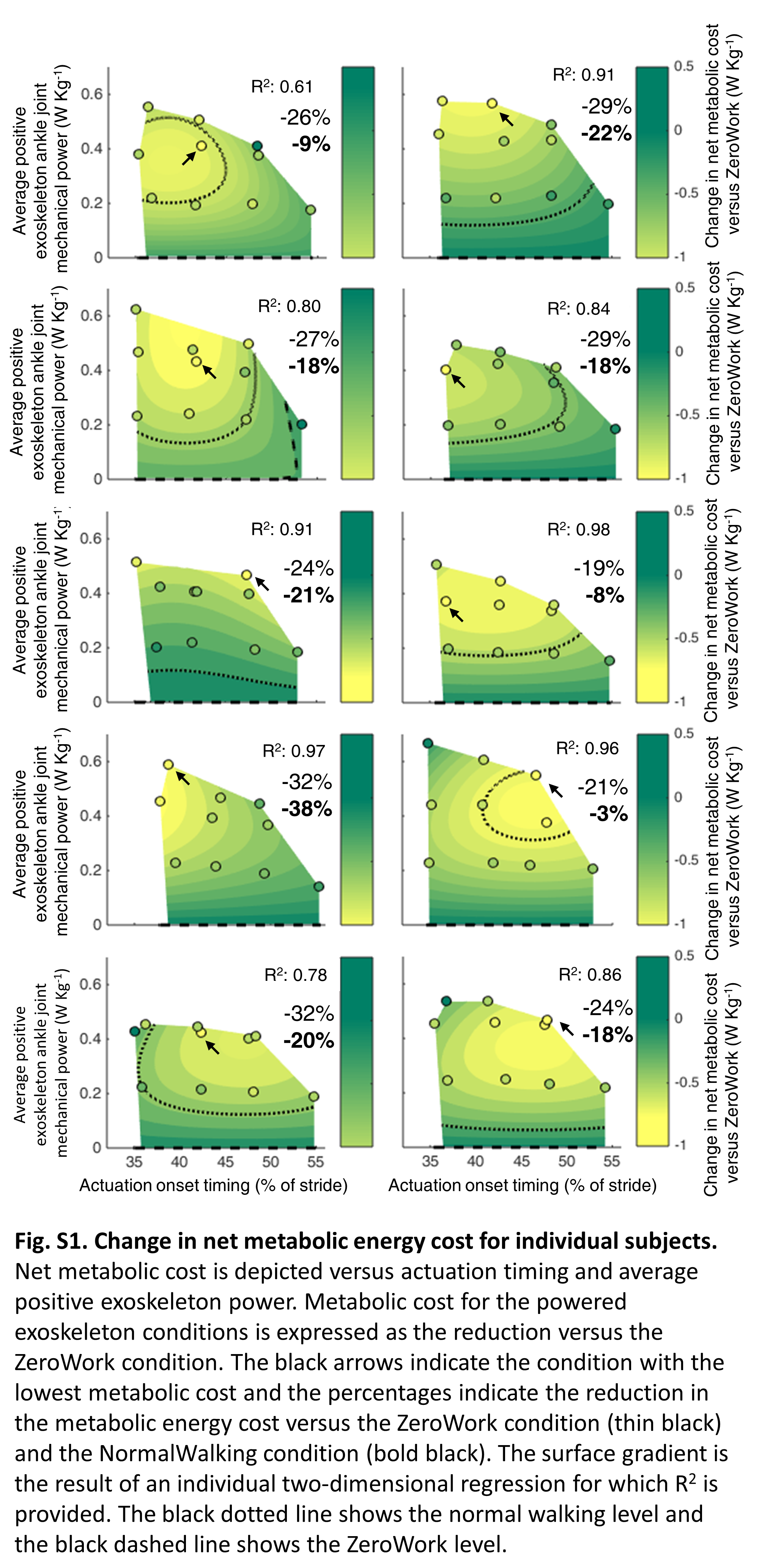

Supplement: Supplementary file 1 — Change in net metabolic energy cost for individual subjects. (TIF 3 mb) [file 12984_2017_235_MOESM1_ESM.tif]

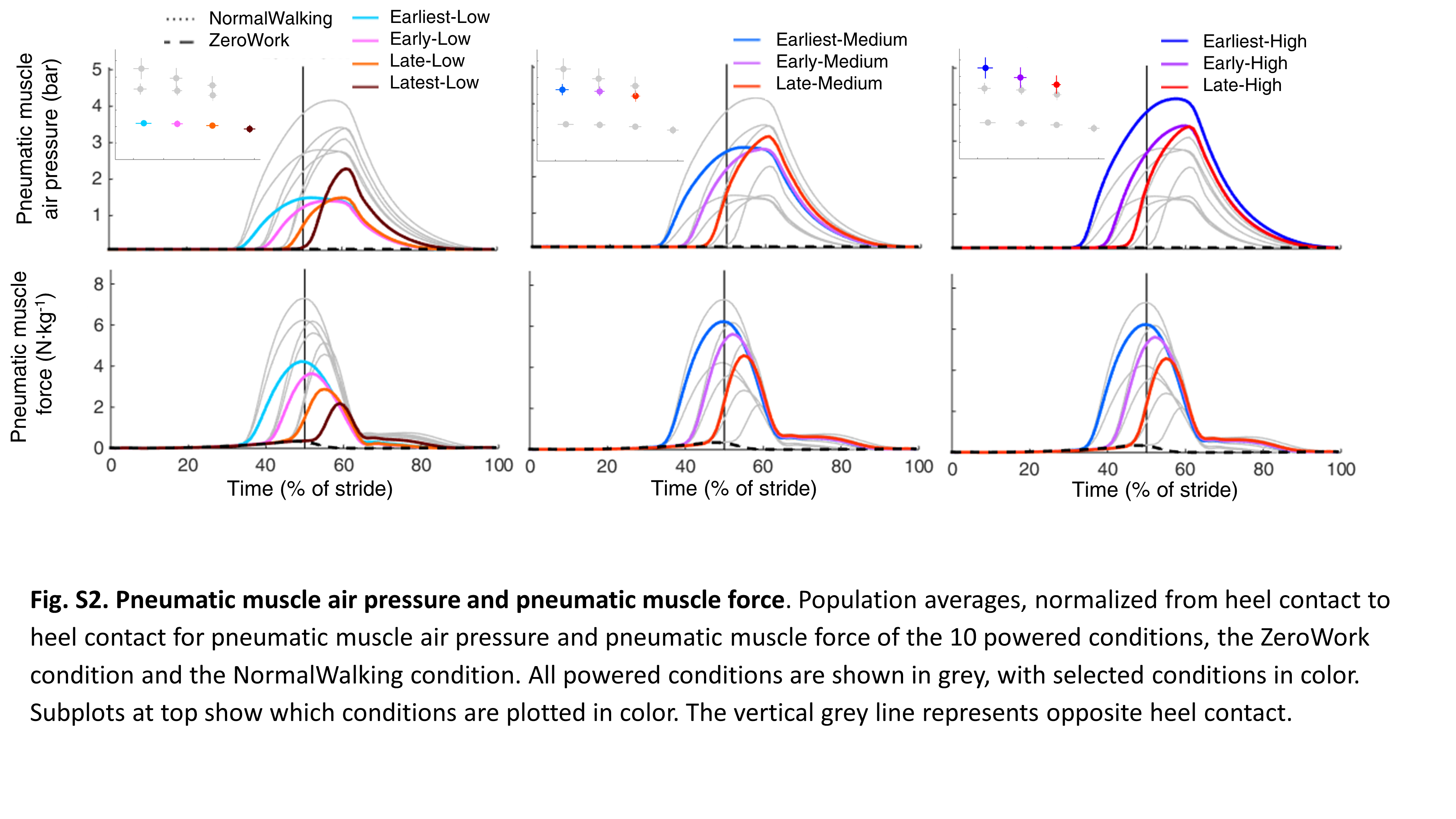

Supplement: Supplementary file 2 — Pneumatic muscle air pressure and pneumatic muscle force. (TIF 2 mb) [file 12984_2017_235_MOESM2_ESM.tif]

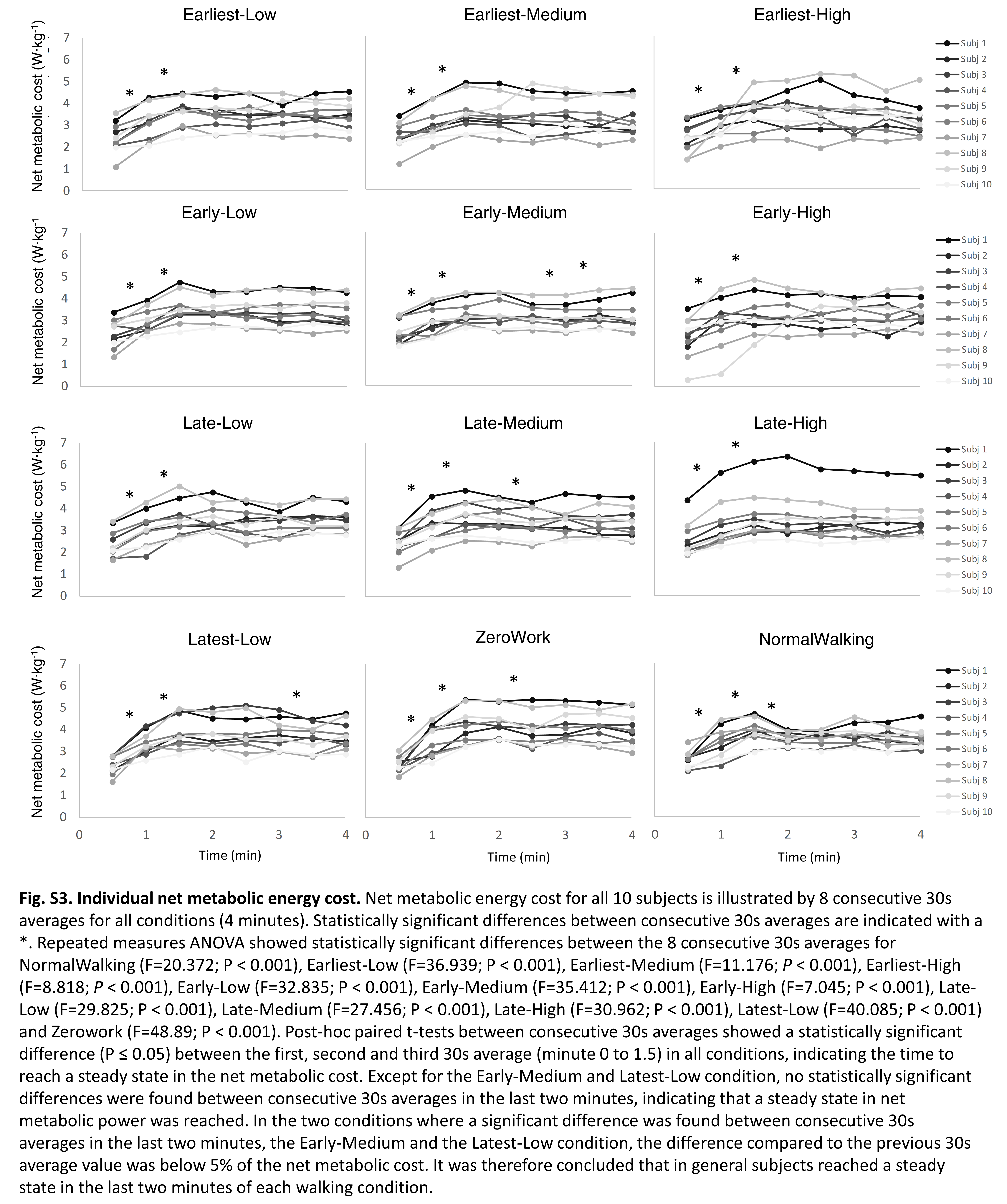

Supplement: Supplementary file 3 — Individual net metabolic energy cost. (TIF 3 mb) [file 12984_2017_235_MOESM3_ESM.tif]

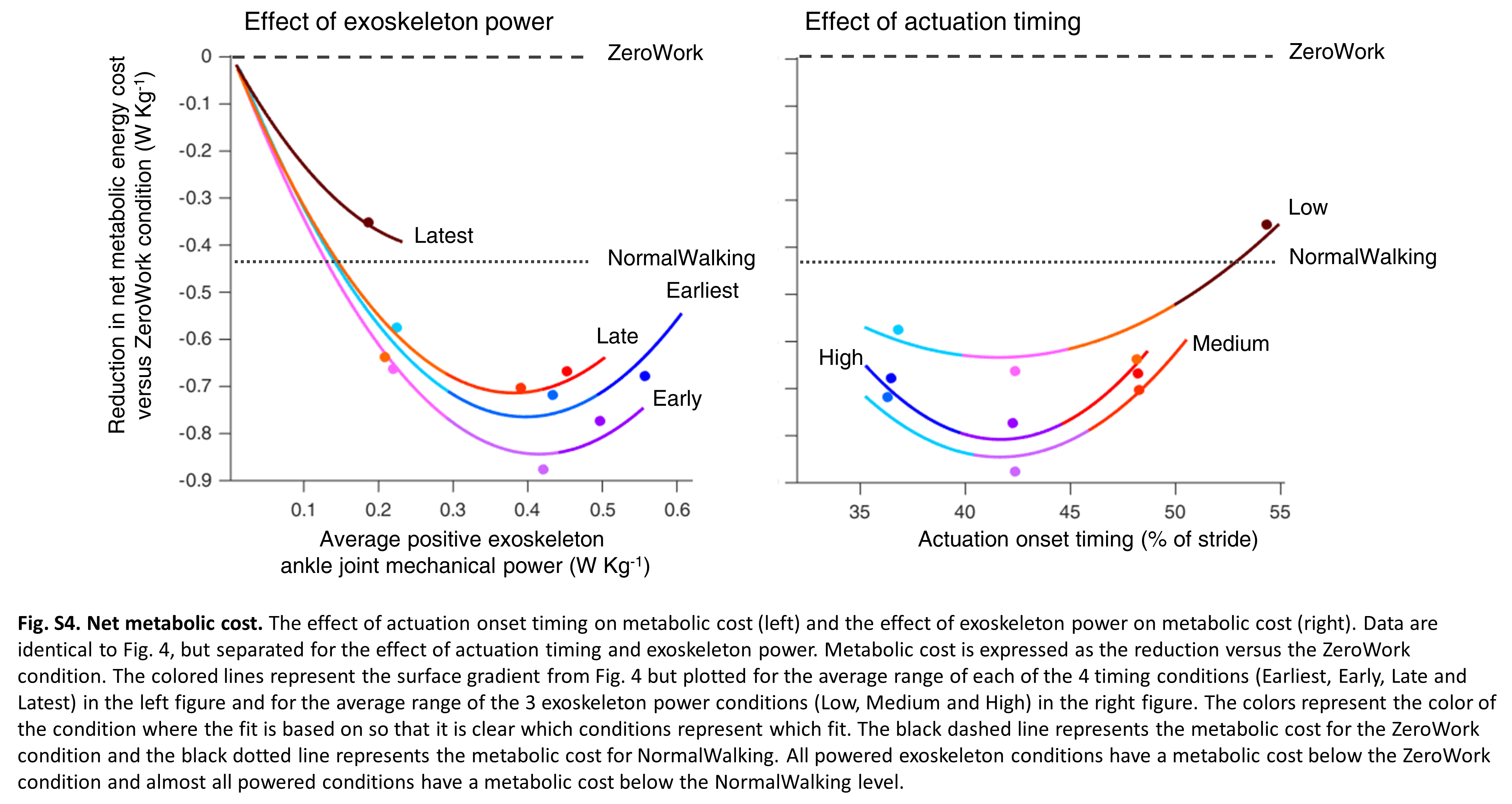

Supplement: Supplementary file 4 — Net metabolic cost. (TIF 1 mb) [file 12984_2017_235_MOESM4_ESM.tif]
